# Dimethyl biphenyl-4,4′-dicarboxyl­ate

**DOI:** 10.1107/S1600536809023605

**Published:** 2009-06-24

**Authors:** Verena Ritzerfeld, Axel Pyrlik, Yutian Wang, Ulli Englert

**Affiliations:** a Institut für Anorganische Chemie, RWTH Aachen, Landoltweg 1, 52074 Aachen, Germany

## Abstract

The asymmetric unit of the title compound, C_16_H_14_O_4_, consists of one half-mol­ecule of an essentially planar biphenyl­dicarboxylic acid ester, with the complete molecule generated by an inversion centre. The maximum deviation from a least-squares plane through all non-H atoms occurs for the peripheric methyl groups and amounts to 0.124 (2) Å. The solid represents a typical mol­ecular crystal without classical hydrogen bonds. The shortest inter­molecular contacts do not differ significantly from the sum of the van der Waals radii of the atoms involved.

## Related literature

For standard van der Waals radii, see: Bondi (1964 ▶). For related structures, see: Li & Brisse (1994 ▶); Marsh & Clemente (2007 ▶); Tashiro *et al.* (1990 ▶).
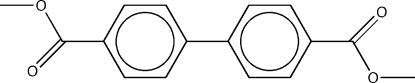


## Experimental

### 

#### Crystal data

C_16_H_14_O_4_
*M* *_r_* = 270.27
Orthorhombic, 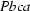

*a* = 7.1358 (9) Å
*b* = 5.9752 (8) Å
*c* = 29.709 (4) Å
*V* = 1266.7 (3) Å^3^
*Z* = 4
Mo *K*α radiation
μ = 0.10 mm^−1^
*T* = 100 K
0.11 × 0.06 × 0.01 mm

#### Data collection

Bruker SMART CCD area-detector diffractometer
Absorption correction: none
14661 measured reflections
1585 independent reflections
1242 reflections with *I* > 2σ(*I*)
*R*_int_ = 0.061

#### Refinement

*R*[*F* ^2^ > 2σ(*F* ^2^)] = 0.050
*wR*(*F* ^2^) = 0.142
*S* = 1.08
1585 reflections
92 parameters
H-atom parameters constrained
Δρ_max_ = 0.46 e Å^−3^
Δρ_min_ = −0.19 e Å^−3^

### 

Data collection: *SMART* (Bruker, 2001 ▶); cell refinement: *SAINT-Plus* (Bruker, 1999 ▶); data reduction: *SAINT-Plus*; program(s) used to solve structure: *SHELXS97* (Sheldrick, 2008 ▶); program(s) used to refine structure: *SHELXL97* (Sheldrick, 2008 ▶); molecular graphics: *PLATON* (Spek 2009 ▶); software used to prepare material for publication: *SHELXL97*.

## Supplementary Material

Crystal structure: contains datablocks I, global. DOI: 10.1107/S1600536809023605/hg2528sup1.cif

Structure factors: contains datablocks I. DOI: 10.1107/S1600536809023605/hg2528Isup2.hkl

Additional supplementary materials:  crystallographic information; 3D view; checkCIF report

## Figures and Tables

**Fig. 1.**
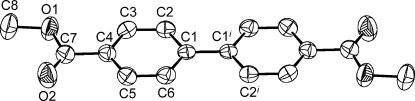
: PLATON (Spek, 2009) plot with displacement ellipsoids at 90% probability; H atoms are not shown. Symmetry code (i) -x + 1,-y,-z.

**Fig. 2.**
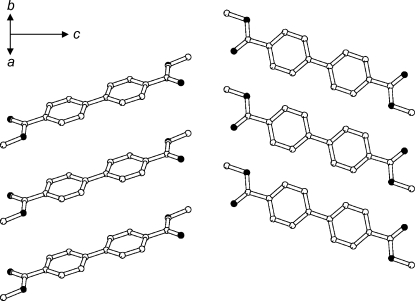
: View of the herringbone like packing (Spek, 2009).

## supplementary crystallographic information

### Comment

In the context of a study devoted to fruit esters, we attempted to enclathrate these compounds as guests into 4,4'-biphenylcarboxylic acid dimethylester as the host structure. In one of these experiments, the potential host was dissolved in boiling ethylacetate and slowly recrystallized. The platelet-shaped crystals obtained did not include any guest molecule but rather enabled us to study the hitherto unknown crystal structure of the pure title compound. Esters of the same acid had been structurally characterized as derivatives of aliphatic (Li & Brisse, 1994) and aromatic alcohols (Tashiro *et al.*, 1990; Marsh & Clemente, 2007). Interesting degrees of freedom in our structure are associated with rotation around the central biphenyl axis and the single bond between the carboxylic C atom C7 (Fig. 1) and the aromatic ring. The former is fixed to planarity for symmetry reasons because the molecules occupy inversion centers in space group *Pbca*. The 1,6 contact between the *ortho* H atoms next to the central C1—C1*^i^* bond is therefore rather short and amounts to 2.02 Å. Interestingly, Tashiro and coworkers (Tashiro *et al.*, 1990) have found both coplanar and non-coplanar biphenyl systems for two different crystalline modifications of the same compound. The second degree of freedom results in a rather small dihedral angle of 6.37 (10) ° subtended by least-squares planes through C1—C6 on the one and C7, C8, O1 and O2 on the other hand. The precise molecular symmetry is therefore *C_i_*, with only small deviations from the supergroup *C_2 h_*. The packing of the molecules reveals a herringbone-like structure as seen in Fig. 2, in which the methyl groups can avoid each other. When standard van-der-Waals radii (C 1.70, H 1.20, O 1.52 Å, Bondi 1964) are taken into account, an overall packing coefficient of 74.3% is calculated (Spek 2009).

### Experimental

About 300 mg (1.1 mmol) of 4,4'-Biphenylcarboxylic acid dimethylester was dissolved in 20 ml of boiling ethylacetate (350 K). The solution was refluxed for about 15 minutes and after hot filtration very slowly (15 h) cooled to 320 K. Several hours later, *ca* 50 mg of platelet-shaped colourless crystals were recovered by filtration.

### Refinement

Hydrogen atoms were included as riding in standard geometry (C_aryl_—H 0.95 Å, C_methyl_—H 0.98 Å).

### Crystal data

**Table:**

C_16_H_14_O_4_	*F*(000) = 568
*M**_r_* = 270.27	*D*_x_ = 1.417 Mg m^−^^3^
Orthorhombic, *P**b**c**a*	Mo *K*α radiation, λ = 0.71073 Å
Hall symbol: -P 2ac 2ab	Cell parameters from 2384 reflections
*a* = 7.1358 (9) Å	θ = 2.7–27.9°
*b* = 5.9752 (8) Å	µ = 0.10 mm^−^^1^
*c* = 29.709 (4) Å	*T* = 100 K
*V* = 1266.7 (3) Å^3^	Plate, colourless
*Z* = 4	0.11 × 0.06 × 0.01 mm

### Data collection

**Table:**

Bruker SMART CCD area-detector diffractometer	1242 reflections with *I* > 2σ(*I*)
Radiation source: fine-focus sealed tube	*R*_int_ = 0.061
graphite	θ_max_ = 28.4°, θ_min_ = 2.7°
ω scans	*h* = −9→9
14661 measured reflections	*k* = −8→7
1585 independent reflections	*l* = −39→39

### Refinement

**Table:**

Refinement on *F*^2^	Primary atom site location: structure-invariant direct methods
Least-squares matrix: full	Secondary atom site location: difference Fourier map
*R*[*F*^2^ > 2σ(*F*^2^)] = 0.050	Hydrogen site location: inferred from neighbouring sites
*wR*(*F*^2^) = 0.142	H-atom parameters constrained
*S* = 1.08	*w* = 1/[σ^2^(*F*_o_^2^) + (0.07*P*)^2^ + 0.4*P*] where *P* = (*F*_o_^2^ + 2*F*_c_^2^)/3
1585 reflections	(Δ/σ)_max_ < 0.001
92 parameters	Δρ_max_ = 0.46 e Å^−^^3^
0 restraints	Δρ_min_ = −0.19 e Å^−^^3^

### Special details

**Table:**

Geometry. All e.s.d.'s (except the e.s.d. in the dihedral angle between two l.s. planes) are estimated using the full covariance matrix. The cell e.s.d.'s are taken into account individually in the estimation of e.s.d.'s in distances, angles and torsion angles; correlations between e.s.d.'s in cell parameters are only used when they are defined by crystal symmetry. An approximate (isotropic) treatment of cell e.s.d.'s is used for estimating e.s.d.'s involving l.s. planes.
Refinement. Refinement of *F*^2^ against ALL reflections. The weighted *R*-factor *wR* and goodness of fit *S* are based on *F*^2^, conventional *R*-factors *R* are based on *F*, with *F* set to zero for negative *F*^2^. The threshold expression of *F*^2^ > σ(*F*^2^) is used only for calculating *R*-factors(gt) *etc*. and is not relevant to the choice of reflections for refinement.

### Fractional atomic coordinates and isotropic or equivalent isotropic displacement parameters (Å^2^)

**Table:**

	*x*	*y*	*z*	*U*_iso_*/*U*_eq_	
O1	0.43822 (17)	0.51545 (19)	0.16205 (4)	0.0236 (3)	
O2	0.55690 (18)	0.1972 (2)	0.19068 (4)	0.0299 (3)	
C1	0.49919 (19)	0.0457 (2)	0.02340 (4)	0.0127 (3)	
C2	0.42227 (19)	0.2566 (2)	0.03299 (5)	0.0152 (3)	
H2	0.3706	0.3428	0.0092	0.018*	
C3	0.41984 (19)	0.3421 (2)	0.07637 (5)	0.0155 (3)	
H3	0.3675	0.4857	0.0819	0.019*	
C4	0.49395 (19)	0.2179 (3)	0.11195 (5)	0.0153 (3)	
C5	0.5698 (2)	0.0075 (2)	0.10315 (5)	0.0171 (3)	
H5	0.6194	−0.0791	0.1272	0.021*	
C6	0.5736 (2)	−0.0766 (2)	0.05963 (5)	0.0165 (3)	
H6	0.6274	−0.2195	0.0542	0.020*	
C7	0.4997 (2)	0.3039 (3)	0.15895 (5)	0.0178 (3)	
C8	0.4498 (3)	0.6139 (3)	0.20658 (6)	0.0271 (4)	
H8A	0.3676	0.5318	0.2272	0.041*	
H8B	0.4104	0.7708	0.2052	0.041*	
H8C	0.5793	0.6055	0.2174	0.041*	

### Atomic displacement parameters (Å^2^)

**Table:**

	*U*^11^	*U*^22^	*U*^33^	*U*^12^	*U*^13^	*U*^23^
O1	0.0335 (7)	0.0195 (6)	0.0177 (6)	0.0065 (5)	−0.0029 (5)	−0.0049 (4)
O2	0.0465 (8)	0.0260 (7)	0.0172 (6)	0.0084 (6)	−0.0047 (5)	0.0001 (5)
C1	0.0095 (6)	0.0129 (7)	0.0157 (7)	−0.0016 (5)	0.0015 (5)	0.0009 (5)
C2	0.0142 (7)	0.0144 (7)	0.0170 (7)	0.0016 (5)	−0.0017 (5)	0.0024 (5)
C3	0.0129 (7)	0.0133 (7)	0.0204 (8)	0.0013 (5)	−0.0006 (5)	−0.0008 (5)
C4	0.0142 (7)	0.0166 (7)	0.0152 (7)	−0.0012 (6)	0.0013 (5)	0.0003 (5)
C5	0.0170 (8)	0.0168 (7)	0.0174 (7)	0.0021 (6)	−0.0009 (6)	0.0023 (5)
C6	0.0172 (7)	0.0131 (7)	0.0193 (7)	0.0028 (5)	0.0009 (6)	0.0005 (5)
C7	0.0180 (8)	0.0181 (8)	0.0173 (7)	−0.0009 (6)	0.0007 (6)	0.0000 (5)
C8	0.0343 (10)	0.0261 (9)	0.0210 (8)	0.0033 (7)	−0.0019 (7)	−0.0092 (7)

### Geometric parameters (Å, °)

**Table:**

O1—C7	1.3409 (19)	C3—H3	0.95
O1—C8	1.4502 (19)	C4—C5	1.394 (2)
O2—C7	1.2093 (18)	C4—C7	1.488 (2)
C1—C2	1.4035 (19)	C5—C6	1.387 (2)
C1—C6	1.4052 (19)	C5—H5	0.95
C1—C1^i^	1.494 (3)	C6—H6	0.95
C2—C3	1.386 (2)	C8—H8A	0.98
C2—H2	0.95	C8—H8B	0.98
C3—C4	1.395 (2)	C8—H8C	0.98
			
C7—O1—C8	115.23 (12)	C6—C5—H5	119.7
C2—C1—C6	117.32 (13)	C4—C5—H5	119.7
C2—C1—C1^i^	121.35 (15)	C5—C6—C1	121.21 (13)
C6—C1—C1^i^	121.33 (16)	C5—C6—H6	119.4
C3—C2—C1	121.64 (13)	C1—C6—H6	119.4
C3—C2—H2	119.2	O2—C7—O1	123.63 (14)
C1—C2—H2	119.2	O2—C7—C4	123.96 (14)
C2—C3—C4	120.23 (13)	O1—C7—C4	112.39 (13)
C2—C3—H3	119.9	O1—C8—H8A	109.5
C4—C3—H3	119.9	O1—C8—H8B	109.5
C5—C4—C3	118.99 (14)	H8A—C8—H8B	109.5
C5—C4—C7	118.47 (13)	O1—C8—H8C	109.5
C3—C4—C7	122.52 (13)	H8A—C8—H8C	109.5
C6—C5—C4	120.60 (14)	H8B—C8—H8C	109.5

Symmetry codes: (i) −*x*+1, −*y*, −*z*.
